# Chronic Bilateral Distal Radial and Ulnar Artery Occlusion Presenting as Peripheral Neuropathy in a Young Adult

**DOI:** 10.1016/j.jaccas.2026.106876

**Published:** 2026-02-05

**Authors:** Reynard Laysandro, Amelia Litmantoro Hidayat, Arcita Hanjani Pramudita

**Affiliations:** a Faculty of Medicine, Department of Internal Medicine, Dr. Cipto Mangunkusumo General Hospital, Universitas Indonesia, Jakarta, Indonesia; b Department of Neurology, National Brain Center Hospital, Jakarta, Indonesia; c Indonesian Cancer Foundation, Jakarta, Indonesia

**Keywords:** duplex ultrasonography, ischemic neuropathy, peripheral artery disease, radial artery occlusion, ulnar artery occlusion

## Abstract

**Background:**

Distal radial and ulnar artery occlusion is a rare condition, especially in the absence of trauma, invasive procedures, vasculitis, or embolic events, and it may mimic neuropathic symptoms, leading to delayed diagnosis.

**Case Summary:**

A 32-year-old woman with uncontrolled hypertension, hypercholesterolemia, and obesity presented with 6 months of intermittent pain, tingling, and discoloration in both hands, which had worsened over the past 2 weeks. Examination showed cold extremities, diminished radial pulses, prolonged capillary refill, oxygen saturation below 94% in the fingers, and skin changes; results on the Michigan Neuropathy Screening Instrument and Neuropathy Disability Score indicated mild neuropathy. Duplex ultrasonography confirmed bilateral narrowing of the distal radial and ulnar arteries. Treatment with nifedipine, aspirin, atorvastatin, captopril, and vitamin B complex, as well as lifestyle modification counseling resulted in improvement within 3 days, including fingertip oxygen saturation rising to 95% and improved arterial lumen diameters.

**Discussion:**

This case demonstrates that upper limb peripheral artery disease may present as neuropathy, underscoring the importance of clinical assessment and duplex ultrasonography for early detection and effective conservative management. Management focuses on risk-factor modification, pharmacological therapy (antiplatelets, statins, antihypertensive agents), and supportive neuropathic care, while revascularization is reserved for refractory or limb-threatening cases.

**Take-Home Messages:**

Chronic bilateral distal radial and ulnar artery occlusion is a rare peripheral artery disease that may cause neuropathy owing to hypoperfusion, inflammation, and microvascular dysfunction. Diagnosis relies on clinical evaluation and duplex ultrasonography. Management centers on conservative therapy, including risk-factor control, antiplatelets, statins, and neuropathy treatment.

## Visual Summary

**Figure:**
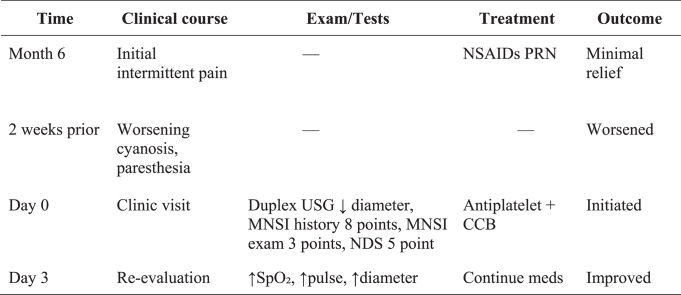

Peripheral artery disease (PAD) is a common cause of morbidity, with an estimated prevalence of 5% to 6% among adults worldwide. Among 236.62 million individuals worldwide, 72.91% of adults aged 25 years and older are found in lower-middle-income countries.^1^^,^^2^ While most epidemiologic attention focuses on lower extremity PAD, clinically important arterial occlusive disease of the upper limbs does occur and may be under-recognized.^1^ Upper limb chronic bilateral distal occlusion involving both the radial and ulnar arteries is uncommon in the published literature and is rarely reported in cohort studies or population surveys. Case reports and case series on bilateral forearm arterial thrombosis or occlusion have underscored the rarity of this condition and the need to recognize atypical causes. Ischemic injury to the peripheral nerves is an established but relatively uncommon mechanism of neuropathy compared with metabolic and inflammatory causes. Pathologic studies of chronically ischemic limbs demonstrate that sustained hypoperfusion produces axonal degeneration, endoneural ischemia, and connective tissue changes in the nerve sheaths that can produce sensorimotor deficits and chronic neuropathic symptoms.^3^

In this report, we present a case of peripheral neuropathy in an adult female patient with chronic bilateral distal radial and ulnar artery occlusion. The patient was treated in a primary health care setting with diagnostic limitations. The case is important for 3 reasons. First, although upper limb PAD and radial/ulnar artery occlusion are described in procedure-related cohorts and registry data, the combination of chronic bilateral distal occlusion of both forearm arteries producing a clinically dominant neuropathy is rarely reported, making this an instructive diagnostic challenge.^4^ Second, the case highlights the pathophysiologic link between chronic ischemia and nerve injury established in neuropathology and clinical series, and it underscores the need to consider vascular imaging when neuropathic symptoms are asymmetrical, painful, or associated with trophic skin changes.^5^ Third, because management options (medical antithrombotic therapy, revascularization, or supportive neuropathic care) depend on accurate diagnosis of the vascular lesion and its chronicity, reporting such atypical presentations adds to the limited evidence base and may prompt more systematic evaluation in similar patients.^6^

## History of Presentation

A 32-year-old woman with a 2-year history of uncontrolled hypertension and hypercholesterolemia presented to a primary care clinic with worsening tingling and refractory pain in both hands. Her symptoms had been intermittent for 6 months but had intensified over the previous 2 weeks, particularly at night, accompanied by a sensation of coldness. The fingers initially appeared pale, progressing to bluish discoloration and chronic intermittent pain. The patient also noticed progressive darkening of both hands, with a sharply demarcated discoloration over the distal third of the forearm.

**Figure 1:**
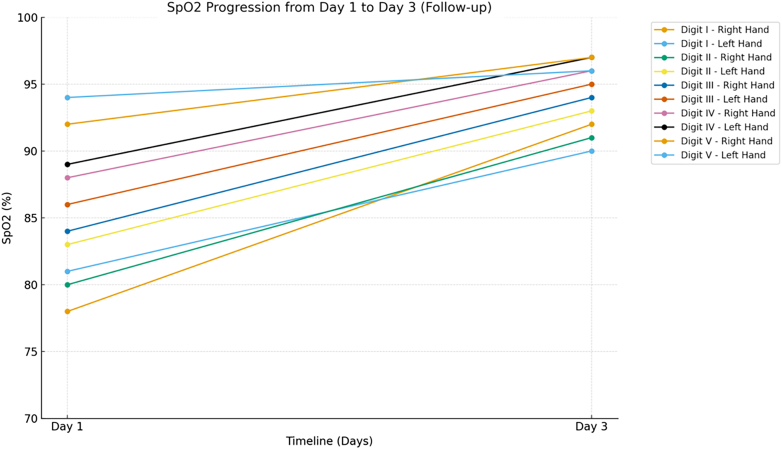
Oxygen Saturation Progression From Day 1 (Pretreatment) to Day 3 (Follow-Up)

**Figure 2:**
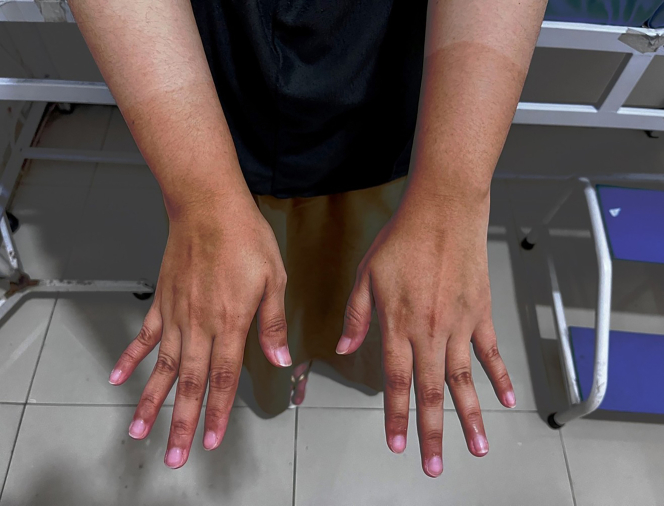
Findings After Physical Examination of the Hands Physical examination revealed darkening skin with a sharply demarcated border over the distal third of the forearm. Mild cyanosis was evident in the fingertips.

On examination, the patient's blood pressure was 160/100 mm Hg with a pulse of 86 beats/min. Her body mass index was 28.3 kg/m^2^. Radial pulses were markedly reduced bilaterally, and ulnar pulses were slightly decreased. All fingers showed oxygen saturation below 94%, capillary refill time of 3 to 4 seconds, and cold temperature (Table 1, Figure 1). Carpal tunnel tests were normal, while the Allen test was abnormal, indicating compromised distal arterial flow. Cardiopulmonary examination was normal. A well-defined darkening of the skin with mild digital cyanosis was evident (Figure 2). Sensory testing revealed reduced light-touch sensation in the darkened areas using a 10-g monofilament. Neuropathy assessments yielded a Michigan Neuropathy Screening Instrument (MNSI) history score of 8, MNSI examination score of 3, and Neuropathy Disability Score of 5, consistent with mild neuropathy.

**Table 1:** Peripheral Oxygen Saturation on Physical Examination of Both Hands

SpO_2_	Day 1 (Pretreatment)		Day 3 (Follow-Up)	
	Right Hand	Left Hand	Right Hand	Left Hand
Digit I	78%	81%	92%	90%
Digit II	80%	83%	91%	93%
Digit III	84%	86%	94%	95%
Digit IV	88%	89%	96%	97%
Digit V	92%	94%	97%	96%

The examination showed increasing oxygen saturation at follow-up (day 3) when compared with the pretreatment condition (day 1).

## Past Medical History

The patient denied dyspnea, palpitations, or symptoms suggestive of cardiopulmonary, rheumatologic, neurological, or vascular disorders, and had no history of infectious diseases such as hepatitis B/C or HIV. She did not smoke or consume alcohol and was taking amlodipine and simvastatin regularly for her hypertension and hypercholesterolemia. She worked as a secretary, performing repetitive hand activities. Family history was significant for type 2 diabetes mellitus and hypertension in her mother.

## Differential Diagnosis

The patient was diagnosed with peripheral neuropathy secondary to chronic bilateral distal radial and ulnar artery occlusion, hypertension grade II, hypercholesterolemia, and obesity.

## Investigations

**Figure 3:**
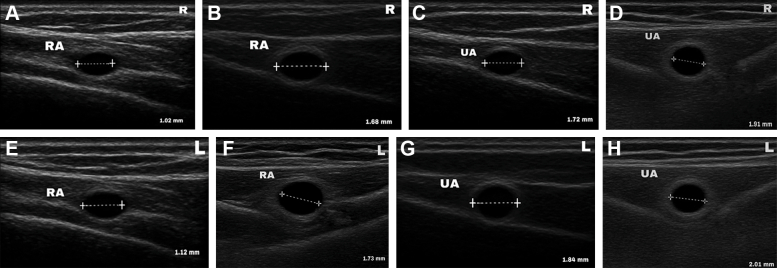
Arterial Ultrasonography Showing the Difference in Radial and Ulnar Artery Size Between Day 1 (Pretreatment) and Day 3 (Follow-Up) Normal sizes are 2.325 ± 0.4 mm (radial artery) and 2.358 ± 0.39 mm (ulnar artery).^7^ (A and B) Comparison of right distal radial artery between (A) pre- and (B) post-treatment showed the artery diameter increase 64.7% after therapy. (C and D) Comparison of right distal ulnar artery between (C) pre- and (D) post-treatment showed the artery diameter increase 11.1% after therapy. (E and F) comparison of left distal radial artery between (E) pre- and (F) post-treatment showed the artery diameter increase 54.5% after therapy. (G and H) comparison of left distal ulnar artery between (G) pre- and (H) post-treatment showed the artery diameter increase 9.24% after therapy. RA = radial artery; UA = ulnar artery.

**Figure 4:**
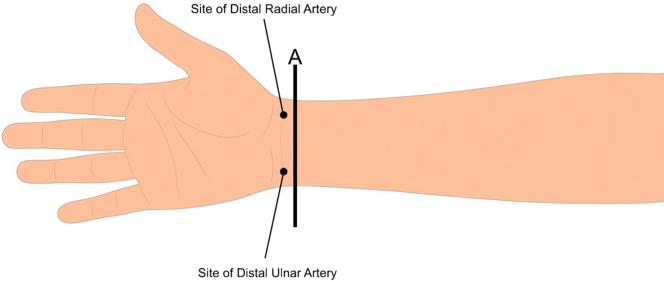
Side of Ultrasonography Application (B-Mode) on the Patient's Arm (Line A)

Laboratory evaluation showed normal complete blood count (hemoglobin: 12.1 g/dL; leukocytes: 6,230/dL; platelets: 260,000/dL; erythrocyte sedimentation rate: 10 mm) and normal fasting blood glucose (118 mg/dL) and uric acid (5.8 mg/dL), with hypercholesterolemia (triglycerides: 221 mg/dL, cholesterol: 248 mg/dL, low-density lipoprotein: 134 mg/dL, high-density lipoprotein: 42 mg/dL). Thyroid-stimulating hormone, folate, and vitamin B12 levels were normal. Duplex ultrasonography (DUS) revealed bilateral narrowing of the distal radial and ulnar arteries (Figures 3 and 4).

## Management

The patient received nifedipine, low-dose aspirin, vitamin B complex, captopril, and atorvastatin, along with lifestyle counseling for weight reduction.

## Outcome and Follow-Up

After 3 days of therapy, symptoms improved, capillary refill time normalized to 2 seconds, and the hands became warm. Peripheral oxygen saturation increased to around 95%, and radial and ulnar pulses strengthened (Table 1, Figure 2). DUS showed improvement in arterial diameters. Vital signs were stable, with blood pressure reduced to 140/90 mm Hg (Figure 3).

## Patient Perspective

Before receiving treatment, the persistent coldness, numbness, and bluish discoloration in both of my hands made daily tasks difficult. I was worried because the symptoms kept returning and I did not know the cause. After undergoing the ultrasound evaluation and starting the prescribed medications, I gradually felt improvement, my hands felt warmer, the color returned, and I could use them more comfortably again. I am grateful for the explanation I received throughout the process and for the careful follow-up. This experience taught me the importance of early medical evaluation and consistency in treatment.

## Discussion

We report the case of a middle-aged woman with chronic bilateral distal radial and ulnar artery occlusion, grade II obesity, and a history of hypertension and hypercholesterolemia. Common causes of such occlusion include atherosclerosis, embolism, trauma, iatrogenic injury (eg, transradial catheterization), repetitive microtrauma, vasculitis, and hypercoagulable states, though atherosclerosis is less frequent in the upper limb arteries. Risk factors typically include older age, smoking, diabetes, hypertension, dyslipidemia, and chronic kidney disease, while small arterial diameter, female sex, and repetitive hand activity also contribute.^8^ In this case, prolonged daily typing combined with hypertension, hypercholesterolemia, and obesity likely played a role, warranting evaluation for traumatic, iatrogenic, vasculitis, or embolic causes.^8^

The pathophysiology of arterial occlusion has many pathways. The immediate mechanism might be caused by thrombosis or embolic obstruction by abrupt reduction of distal perfusion pressure. Acute occlusion causes ischemia, hypoxia, and metabolic failure, while chronic/subacute occlusion causes progressive hypoperfusion, collateral remodeling (incomplete compensation), chronic inflammation, oxidative stress, and microvascular dysfunction. Chronic ischemia also causes muscle atrophy, skin trophic changes, and nerve ischemia. At the cellular level, inflammation induces the expression of VCAM-1, ICAM-1, and E- and P-selections on endothelial cells, enhancing leukocyte–endothelial adhesion and being associated with disease severity.^4^^,^^9^

Peripheral nerves rely on small intraneural vessels; reduced perfusion leads to axonal ischemia, causing degeneration or demyelination. Ischemia induces inflammation, oxidative stress, and sometimes reperfusion injury, resulting in neuropathic pain, paresthesia, and sensory-motor deficits. Chronic hypoperfusion produces chronic axonopathy with poor regeneration, as seen in chronic limb ischemia or ischemic monomelic neuropathy. Clinically, symptoms are mainly painful sensory disturbances with possible motor weakness.^5^ Hyperpigmentation is uncommon but may occur because of microvascular damage and repeated microhemorrhages, causing hemosiderin deposition and chronic inflammation.^7^ Typical ischemic signs include pallor, atrophy, thin shiny skin, hair loss, and ulceration.^6^

We describe a patient with progressive neuropathic pain that had worsened over 2 weeks, with a 6-month history of refractory symptoms. Physical examination revealed decreased pulses, prolonged capillary refill time, cold extremities, hyperpigmented skin with cyanotic nails, and reduced sensory response on the monofilament-10 g test (confirmed by the Semmes-Weinstein test). The examination was conducted in a 26 °C room. Neuropathy screening showed an MNSI (history) score of 8 points, MNSI (examination) score of 3 points, and a Neuropathy Disability Score of 5 points, all suggesting mild neuropathy. Based on this case, it is recommended to first evaluate the history (acute vs chronic onset), pulses, capillary refill, skin or trophic changes, and perform a focused neurological examination assessing sensory and motor deficits. A bedside Allen test should be performed to evaluate hand circulation for radial or ulnar artery involvement. According to the 2024 European Society of Cardiology PAD diagnostic algorithm, evaluation should not be limited to clinical assessment but also include walking impairment assessment (using questionnaires or treadmill testing) and functional assessment such as the Short Physical Performance Battery or the 6-minute walking test. These assessments follow hemodynamic or vascular evaluation, including ankle-brachial index, ankle pressure, toe pressure, transcutaneous oxygen pressure, or DUS, along with wound assessment to detect arterial occlusion.^7^

For hemodynamic or vascular evaluation in this case, B-mode ultrasonography was used to detect anatomical abnormalities. Reference studies recommend duplex (arterial) ultrasound as the first-line noninvasive test to localize stenosis or occlusion and assess blood flow, while ankle-brachial index and plethysmography are more useful for lower extremity disease. Cross-sectional imaging such as computed tomography angiography or contrast-enhanced magnetic resonance angiography provides high accuracy for interventional planning. The gold standard remains digital subtraction angiography, especially when endovascular therapy is considered. However, computed tomography angiography, magnetic resonance angiography, and DUS are used first as less-invasive options.^6^ Nerve conduction studies are the gold standard to confirm neuropathy, assessing large-myelinated fiber function and differentiating demyelinating from axonal abnormalities and symmetric polyneuropathy from focal entrapment.^10^ In the current case, neuropathy was concluded based on examination and screening scores because a nerve conduction study was not available in the primary care setting.

DUS provides high diagnostic accuracy for peripheral arterial disease, with a sensitivity around 86% and a specificity about 95% for detecting >50% stenosis. As a first-line, noninvasive test, it is practical for trained clinicians in primary care or community vascular clinics. Its limitations include operator dependence and reduced sensitivity in very distal or small arteries, where visualization is poor. When DUS is inconclusive or intervention is planned, computed tomography angiography, magnetic resonance angiography, or digital subtraction angiography is indicated. Although angiography is the gold standard, DUS was selected in this case given resource limitations, highlighting the need for strong clinical suspicion and ultrasonography skills in primary care.^6^^,^^7^

Our patient was stable and managed conservatively using nifedipine, aspirin, vitamin B complex, captopril, and atorvastatin to address arterial occlusion, neuropathic symptoms, hypertension, and hypercholesterolemia. For subacute or chronic upper limb occlusions, conservative therapy is strongly recommended, including risk-factor modification, antiplatelet therapy, and statins. If an embolic source is suspected, cardiac evaluation and anticoagulation may be required. Revascularization such as angioplasty, stenting, or surgical bypass is reserved for patients with persistent symptoms, tissue loss, or failure of medical therapy. Radial or ulnar occlusions after transradial catheterization are often managed conservatively, with some cases recanalizing spontaneously.^6^

Neuropathic pain management includes medications such as gabapentin, serotonin and norepinephrine reuptake inhibitors, or tricyclic antidepressants, along with physical therapy and neurology referral. If neuropathy is secondary to ischemia, revascularization may improve nerve function. Supportive skin care, ulcer management, and dermatology input may be needed. Multidisciplinary care involving vascular specialists, internists, neurologists, dermatologists, and rehabilitation teams is essential to address comorbidities, limb-threat severity, and underlying causes.^6^^,^^7^

### Study Limitations

Several limitations are inherent to this case report. First, it describes only the initial standard therapy administered to the patient. Second, there is no long-term follow-up, which limits understanding of the sustained impact of the treatment provided. Third, the patient's risk factors were not comprehensively evaluated given the limited availability of laboratory investigations. Finally, no gold-standard diagnostic test, such as invasive angiography or nerve conduction study, was performed to confirmed arterial occlusion and neuropathy. Future studies are warranted to better understand the clinical implications of chronic bilateral distal upper limb occlusion and to inform strategies for improving patient care.

## Conclusions

Chronic bilateral distal radial and ulnar artery occlusion is a rare form of PAD associated with risk factors such as older age, smoking, diabetes, hypertension, dyslipidemia, and chronic kidney disease, and it may present with peripheral neuropathy due to progressive hypoperfusion, chronic inflammation, oxidative stress, and microvascular dysfunction, leading to chronic axonopathy. Diagnosis relies on careful clinical evaluation and DUS, while management focuses on conservative therapy, including risk-factor modification, antiplatelet and statin therapy, and neuropathy treatment.

### Data Availability

All data supporting the conclusions of this case report are included within the article. Additional anonymized clinical data may be made available by the corresponding author upon reasonable request, in accordance with institutional policies and patient privacy regulations.

### Statement of Consent and Ethics

Written informed consent was obtained from the patient for participation and publication of this case, including clinical information and images. All identifying details were removed to ensure anonymity. This case report was conducted in accordance with the ethical standards of the Declaration of Helsinki, COPE guidance, and CARE guidelines for case reports. According to institutional policy, single-patient case reports do not require formal ethics committee approval.

## Funding Support and Author Disclosures

The authors have reported that they have no relationships relevant to the contents of this paper to disclose.

**Take-Home Messages**

• Chronic bilateral distal radial and ulnar artery occlusion is a rare PAD that may cause neuropathy owing to hypoperfusion, inflammation, and microvascular dysfunction. Diagnosis relies on clinical evaluation and duplex ultrasonography.
• Management centers on conservative therapy, including risk-factor control, antiplatelets, statins, and neuropathy treatment.

**Visual Summary:** Timeline of the Case

Time	Clinical Course	Examination/Tests	Treatment	Outcome
6 Mo prior	Initial intermittent pain	—	NSAIDs as needed	Minimal relief
2 Wk prior	Worsening cyanosis, paresthesia	—	—	Worsened
Day 0	Clinic visit	DUS: ↓arterial diameter; MNSI history score: 8 points, MNSI examination score: 3 points, Neuropathy Disability Score: 5 points	Antiplatelet + CCB	Initiated
Day 3	Re-evaluation	↑SpO_2_, ↑pulse, ↑arterial diameter	Continue medication	Improved

CCB = calcium-channel blocker; DUS = duplex ultrasonography; MNSI = Michigan Neuropathy Screening Instrument; NSAID = nonsteroidal anti-inflammatory drug.
